# The long and winding road to happiness: A randomized controlled trial and cost-effectiveness analysis of a positive psychology intervention for lonely people with health problems and a low socio-economic status

**DOI:** 10.1186/s12955-020-01416-x

**Published:** 2020-06-02

**Authors:** Laura A. Weiss, Martijn A. H. Oude Voshaar, Ernst T. Bohlmeijer, Gerben J. Westerhof

**Affiliations:** 1 Faculty of Behavioural, Management & Social Sciences, Department of Psychology, Health & Technology, Centre for eHealth and Wellbeing Research, University of Twente, Postbus 217, 7500 AE Enschede, The Netherlands; 2 Optentia Research Focus Area, North-West University, Hendrik Van Eck Blvd, Vanderbijlpark, 1900 South Africa

**Keywords:** Randomized controlled trial (RCT) - positive psychology intervention - well-being - loneliness - health problems - social work - flourishing - mental health - depression, Public health

## Abstract

**Background:**

Our objective was to evaluate the effectiveness and cost-effectiveness of the positive psychology intervention ‘Happiness Route’ compared to an active control condition in a vulnerable population with an accumulation of health and psychosocial problems.

**Methods:**

We conducted a randomized, single-blind, actively-controlled, parallel group study in seven municipalities in the Netherlands. To be eligible, participants had to experience loneliness, health problems and low socio-economic status. Each group received several home visits by a counsellor (two in the control condition, two to six in the experimental condition). In the Happiness Route, a happiness-based approach was used, whereas the control condition used a traditional problem-based approach. The primary outcome was well-being, measured with the Mental Health Continuum-Short Form (MHC-SF).

**Results:**

Fifty-eight participants were randomized to the Happiness Route, 50 to the control condition. Participants were severely lonely, had on average three health problems and less than 5% had paid work. The total MHC-SF score, emotional and social well-being, depression and loneliness improved significantly over the nine-month period in both conditions (*p* < .05), but there were no significant changes between the conditions across time. Languishing decreased significantly from 33% at baseline to 16% at follow-up among the Happiness Route participants but did not change significantly in the control condition. No significant improvement over time was found in psychological well-being, resilience, purpose in life, health-related quality of life and social participation. Cost-effectiveness analysis showed that expected saved costs per QALY lost was €219,948 for the Happiness Route, relative to the control condition. The probability was 83% that the Happiness Route was cost saving and 54% that the Happiness Route was cost-effective at a willingness to accept a threshold of €100,000.

**Conclusions:**

Mental health status of both groups improved considerably. However, we could not demonstrate that the Happiness Route yielded better health outcomes compared to the control condition. Nevertheless, the results of the cost-effectiveness analysis suggested that the Happiness Route is an acceptable intervention from a health-economic point of view. Our results should be viewed in light of the fact that we could not include the planned number of participants.

**Trial registration:**

Netherlands Trial Register: NTR3377. Registered 2 Apr 2012.

## Introduction

This study, a randomized controlled trial of the ‘Happiness Route,’ a positive psychology intervention, specifically focuses on a vulnerable group that is suffering from an accumulation of difficulties. Vulnerable adults are defined as individuals who are susceptible to harm [1]. Their vulnerability is the result of the complex interaction between a lack of available resources and the challenges they have to face in their lives. Our target group has a disadvantaged socio-economic status (SES) as well as a lack of a social support network (leading to increased levels of loneliness), combined with personal limitations in the form of health problems. Each of these factors alone – a low SES, loneliness and health problems – is a risk factor for diminished well-being [2–9]. In addition to the negative effects of these risk factors on the individual, these factors can also result in negative societal outcomes, such as the growing costs associated with loneliness [10]. Low SES and loneliness can also lead to a greater risk of early mortality [11] and increased morbidity [12] and are associated with physical illness [13] and mental disorders such as depression [14]. Such an accumulation can precipitate a negative downward spiral, resulting in a serious loss of well-being and an exacerbation of symptoms of (mental) health complaints [15, 16], consequently leading to more health care consumption and higher economic costs.

In this study, rather than treating symptoms, we examined an approach that is based on the principles of positive psychology directed towards improving the well-being of this group of vulnerable adults. The field of positive psychology has expanded traditional psychology by including and examining topics such as strengths, growth and well-being [17]. The promotion of well-being is now widely recognized as a new goal in mental health care to complement the traditional focus on preventing and treating problems [2, 18, 19]. Improved well-being has positive effects on health and personal functioning, resulting in health gains at both the individual and societal level [20].

Several meta-analyses show that positive psychology interventions (PPIs) can improve emotional and psychological well-being [21–23] . However, despite earlier findings that PPIs tend to yield better results in these populations [20, 21], only a minority of intervention studies target groups with multiple health and psychosocial problems. Studies addressing clinical populations were all directed to groups with a specific problem, either a physical or mental disorder (e.g., cancer or depression), a problematic condition (e.g., loneliness), or to a specific age group (e.g., the elderly). Yet many people suffer from multiple problems and diseases making them especially vulnerable [24]. Given this population’s high level of suffering and the resultant negative implications for society, PPIs need to be more widely used among groups with more complex vulnerabilities. Therefore, we evaluated a PPI for a group with a complex of problems spanning across their lifespan, rather than for a target group with a specific disease or condition.

One of the few happiness-based interventions that has been successfully implemented in practice in local communities in the Netherlands [25] is called the ‘Happiness Route’ [26]. To evaluate the intervention, this study uses a multicentre trial design and tests the intervention the way it is delivered in the everyday practice of social work, comparing it to an active control condition. One of the goals of our study, therefore, was to add valuable theoretically and practical information with regard to whether PPIs can improve well-being in people with great vulnerabilities and how the PPIs might do so.

The aims of this randomized controlled study were to examine (1) *reach*: the characteristics of the reached participants; (2) *effectiveness*: effects of the Happiness Route intervention in comparison to Customized Care regarding the (a) primary outcome measure of well-being and the (b) secondary outcome measures of resilience, purpose in life, depression, health-related quality of life, loneliness, social participation and health care costs; (3) *treatment satisfaction* of the participants, comparing the Happiness Route intervention to Customized Care; and (4) *cost-effectiveness*: to compare the cost-effectiveness of the Happiness Route with the control condition.

## Methods

### Design

The trial, conducted in the Netherlands, was a multicentre, equally randomized, parallel group study with an experimental condition and active control condition. The randomization with participants as unit had an allocation ratio of 1:1 for the two groups. Only participants were blinded to the expectations with regard to both conditions. A detailed description of the study design is published in a research protocol [27]. Instead of the ten centres that were originally planned, seven centres took part, as three centres dropped out. Also, the number and distribution of participants across the centres did not correspond to the original plan, as the inclusion of participants was challenging and progressed differently between each centre.

### Participants

#### Recruitment and setting

Intermediaries who were regularly in contact with the target group during their daily work recruited the participants. These intermediaries were professionals from the health and welfare sector, such as social workers, general practitioners, nurses, or people working in home care. The counsellors of both the experimental and control condition could also propose candidates. The project leader of the municipality identified and informed the intermediaries. Data was collected in seven local municipalities throughout the Netherlands. The population of the municipalities ranged from circa 33,000 (Tynaarlo) to 176,000 inhabitants (Nijmegen). The seven project leaders were located in a local municipality (Hengelo), a foundation that supports leisure time for people with a disability (Almelo), a service club (Woerden), a mental health care organization (Nijmegen) and welfare organizations (Zeist, Tynaarlo, Assen). Two of the project leaders were volunteers (Woerden and Zeist).

#### Inclusion and exclusion criteria

Eligible participants were aged 18 or older; experiencing loneliness (scoring 3 or higher on the loneliness scale [28]); suffering from mental and/or physical health problems (having at least one health limitations in the EuroQol 5D (EQ-5D) questionnaire [29]); and with a low socio-economic status, which was defined as having a low education (no more than a lower secondary education) and/or low employment status (not having a paid job) and/or low financial income (as defined by the Statistics Netherlands [30]: less than €1000 per month for a single household and between €1000 and €1500 for a couple or a single household with at least one child).

Candidates were excluded if they met one or more of the following criteria: high well-being (one standard deviation above the mean of the Dutch population on the Mental Health Continuum-Short Form (MHC-SF), that is, a score of 4.83 or higher [31]); severe, untreated depression (scoring 39 or higher on the Center for Epidemiology Depression Scale (CES-D), without being under psychological treatment [32, 33]); being currently in a crisis situation, such as being homeless or having recently lost a partner (judged by the counsellor during the intake); or insufficient cognitive or linguistic skills to fill out a questionnaire (judged by the counsellor during the intake).

#### Sample size

A small to medium effect size was expected for the Happiness Route condition (Cohen’s d = .35), based on a meta-analysis on positive psychology interventions [22]. Preceding the study, the G*power program was employed to determine that a total sample size of 204 would be required to have an 80% chance to detect an effect of this magnitude at α = .05 (one-sided). To account for a maximum of 20% drop out, 256 participants were to be included – 128 in each condition. However, the study could not be completed with the sample size and power originally planned. Instead, 108 participants were included, 58 receiving the Happiness Route and 50 Customized Care. None of the municipalities could recruit the intended number of participants (Hengelo *n* = 43, Almelo *n* = 26, Zeist *n* = 21, Woerden *n* = 8, Nijmegen *n* = 4, Tynaarlo *n* = 3, Assen *n* = 3). When looking at the realized power, there was a 56% chance to obtain statistically significant results with an effect size of 0.35, as calculated with the G*power program.

#### Randomization and blinding

Eligible participants were randomized to either the Happiness Route or Customized Care. The random allocation sequence was generated a priori by a computer-generated randomized number list, made with randomizer.org. No restriction or stratification was used. The list was concealed and only used when the next participant had to be assigned to one of the two groups. The first author, LAW, generated the random allocation sequence. Enrolling and assigning participants was performed by LAW, and partly by a student assistant (PH) after receiving thorough training and while under supervision. The project leaders and counsellors were not involved in the sequence generation and allocation concealment in any way. LAW or PH informed the local project leader about the outcome of the inclusion and randomization, who then appointed a suitable counsellor to the participant, if possible, the same counsellor who conducted the intake. The counsellor informed the participant about which group he or she was randomized to and delivered the intervention during the following 3 months.

Those who were aware of the allocated condition included the project leaders, counsellors who delivered the interventions and partially helped to collect data, investigators who assessed the outcomes and the researchers who conducted the statistical analysis. Participants were kept blinded to the expectations related to their allocation. They were informed that they would be randomly assigned to one of two groups and that each group had a different approach to delivering optimal care. After the inclusion was completed, participants were told to which group they were assigned. They were not informed that one group was a control condition while the other group was the intervention that under investigation. These terms mean that, although participants were not fully blinded to the intervention, they were blinded to the expectations and the fact that an experimental condition was compared to a control condition. None of the participants had to be unblinded while the trial was being conducted.

### Interventions

#### Counsellors

Of the 66 trained counsellors, 52 were professionals from the health care and welfare sector and 14 were experienced volunteers. All counsellors received intensive training on how to conduct the research and intervention by the main researcher (LAW): 46 were trained to deliver the Happiness Route and 20 to deliver the control condition. Volunteers were all in the Happiness Route condition. All counsellors received regular (peer) intervision (about twice a year), supervised by LAW. Counsellors had a mean age of 50.09 years (SD 12.81), ranging between 23 and 70 years. Eighty-three percent of the counsellors were female, 92% were Dutch and 93% followed higher education, while 7% had a medium education level. They had a mean of 18 years (SD 12.61) work experience in the health care or welfare sector, with a maximum experience of 43 years.

#### Experimental condition: the happiness route

The Happiness Route has been developed in the municipality of Almelo in the Netherlands and was implemented by the non-profit organization Arcon in multiple cities throughout the region. Adapted and formalized by the University of Twente to be more theory-driven, the Happiness Route is a positive psychology intervention [34], using the principles of self-determination theory [35] as it supports the autonomy, competence and relatedness of participants. The aim of the intervention is to increase well-being by supporting participants to find and act on a passion or intrinsically motivated activity.

Over the course of 3 months, counsellors visited participants at home between two to six times. The time interval between visits depended on the wishes and needs of the individual participant, but generally visit intervals were between 1 to 3 weeks. Practice has shown that the number of home visits can vary between participants, as they differ widely in how quickly they can identify a passion and their need for support. A session had a maximum duration of 90 min. After shortly talking about the participant’s current situation, problems were explicitly ‘laid aside’. Instead, the counsellor asked questions that aimed to discover sources of happiness for the participant, such as: ‘What makes your eyes twinkle?’ The counsellor could choose from a set of evidence-based methods that he or she considered best for the individual and situation, such as examining values (‘What is important to you?’), behavioural activation [36] or life-review methods [31] (looking back on their childhood and reflecting on happy memories, e.g. ‘Which dreams did you have as a child? Who was your childhood hero? What was your dream job? During which kind of activities did you have fun?’). Counsellors would also encourage participants to examine future dreams, with questions such as: ‘What would you do if nothing could hold you back? Is there something you always wanted to do, but you never did?’

To explore all options, a list of all named interests, dreams and possibilities was written down. Finally, the participant had to choose an activity to carry out. This could be supported by the method of ‘anticipated regret’; imagining what you would regret most if you had not done it in a couple of years (see [27]). The choice of activity needed to be related to the passion of the participant. The participant had the final say in the chosen activity while counsellors only offered support to make sure that the choice could be done autonomously. For example, if someone’s passion was art, the chosen activity might be taking painting classes. The counsellors were trained to not exclude any activity in the beginning, but to later assess whether the activity could be realized and, if necessary, gently nudge the participant towards a more realistic activity.

Counsellors supported participants in the process of finding an activity and acting upon it, but participants were explicitly encouraged to actively search and plan a preferably long-lasting activity. Possible pitfalls and how to deal with them were discussed. The choice of activity needed to be made by the participant in order to strengthen feelings of autonomy. Ideally, existing talents could be used or skills could be developed during the activity, in order to strengthen feelings of competence. If the participant was able to come into contact with others during the chosen activity, it was also an advantage to strengthening feelings of relatedness. However, none of these particulars were explicitly demanded. The only requirement was that the participant had to spend the budget on him/herself to buy something that was related to a passion or intrinsically motivated activity.

The participants were allowed to spend up to €500 to realize their passion. The money could be used to pay for an activity (e.g., a painting course or yoga classes) or to purchase something needed for an activity (e.g., a camera or a season ticket to the local football club). When a suitable activity was found, a budget application form was filled out together by the counsellor and participant and then sent to the project leader. The project leader decided if the activity fit the passion and usually sent the money directly to the account of the participant. LAW and AF trained the project leaders on how to identify if an activity was suitable. They would especially focus on one question in the form in which the counsellor had to explain how the passion was linked to the activity. If that was not sufficiently clear, the project leader would refer back to the counsellor and ask for clarification. We also provided the opportunity to discuss decisions with the lead researcher. We additionally provided the project leader with the option to consult with a committee (with experienced former project leaders). The money was not always needed, for example, when people chose to engage in voluntary work as an activity.

The aim was to find an activity and start doing it within 3 months. The last session was an early feedback session, so that first experiences could be shared, the activity could be evaluated and, if needed, adjusted. The counsellor could also evaluate if the money was spent in the intended way. Also, the project leaders would check sporadically on random cases, to ensure that the money was used as described in the application. In practice, some participants started performing the activity after 3 months, often due to practical reasons, such as the starting date of a course.

We ensured the integrity of the intervention by giving all counsellors an intensive training of one to two full days, where we practised the intervention with exercises and role-plays. Also, intervision sessions (led by the LAW) were organized at least every 6 months, where questions could be asked and experiences exchanged with fellow counsellors, the researcher or the project leader. During intervision sessions, the group members discussed all of their current cases.

#### Control condition: customized care

The active control condition, called `Customized Care´, encompassed two home visits with a maximum duration of 90 min by a health care professional, over a time span of 3 months. In this group, no volunteers were used, as they did not have the authority to implement, optimise or change health or social services being provided to the participant. The aim of this active control group was to provide the participants of the control condition with professional attention, similar to the experimental condition. This way, we could control for nonspecific treatment effects (attention, time and the expectation to receive help). A further goal was to provide the participants of the control condition with the best possible care through the traditional problem-focus approach.

During the first session, counsellor and participant worked towards a shared definition of what the participant’s problems entailed. Next, participants were asked how satisfied they were with the care they were receiving, taking stock of all the services they used with the help of a list. Possible mismatches between needs and received care could be identified at this stage. In the cases where participants were either dissatisfied, needed help for certain problems or received abundant help, the counsellor tried to improve the situation. Adjustments to care included any form of help the counsellor considered appropriate, such as organizing more or less care or different care. Possible options for optimizing the care situation were discussed with the participant, with the counsellor taking the lead and informing the participant about all possible options of services that might be unknown to the participant. Counsellors ensured that each participant received the best possible care and optimised the care, if needed. Beforehand, it was estimated that 3 h would be enough to work through all these steps, but practice showed that, in a couple of cases, one or two more sessions were needed. Any possible changes to a participant’s care were supposed to start within 3 months. If the care was already optimal, nothing needed to be changed, but the participant was assured to receive the best possible care available. Examples of possible changes were more support for a certain area or task (e.g., domestic help), counselling, or other forms of therapy (e.g., occupational therapy). After having filled out the last questionnaire, participants of the control condition were debriefed and then given the opportunity to take part in the Happiness Route as well.

### Procedure and materials

An intermediary could apply for a candidate that seemed eligible by sending an application form, signed by the candidate, to the project leader. The project leader checked the application form and sent either a Happiness Route or Customized Care counsellor to do an intake. Beforehand, the candidate received a letter with information about the aim of the project, eligibility, what it meant to participate, how the project worked, the benefits of participation, information about how participation was voluntary, what would happen with the data, and whom to contact with questions. During intake, the research-project was explained in more detail and, if the individual was willing to participate, the candidate signed an informed consent and filled out the baseline questionnaire. If needed, the counsellor provided assistance. The informed consent and questionnaire were then sent to the primary investigator (LAW).

### Outcome measures

A paper and pencil questionnaire was used at three time points: at baseline, after 3 months, and 9 months after baseline (follow-up). The same questionnaire with 92 items was used for all three time points, with the exception that the first one, filled out during the intake, also included 13 questions on personal characteristics, and the last two questionnaires included 4 questions to evaluate the intervention. Participants filled out the questionnaires by themselves at home. At baseline, the counsellor was always present and could help. The second and third questionnaires were sent by post. If the participants needed help filling out the questionnaires, they could ask the counsellor for help. If the questionnaire was not sent back after 2 weeks, the investigator contacted the counsellor and requested that he or she check on the participant and help with the questionnaire’s completion, if needed.

#### Primary outcome

The primary outcome of this study was well-being, measured with the Dutch MHC-SF [31]. The scale has 14 items and 3 subscales: emotional well-being (items 1–3), social well-being (items 4–8) and psychological well-being (items 9–14). Emotional well-being is related to the subjective evaluation of one’s well-being in terms of happiness, interest in life and life satisfaction. Social well-being refers to one’s social functioning (e.g., integrating into and contributing to a group), while psychological well-being relates to positive functioning on an individual level (e.g., having a purpose and feeling autonomous). Participants were asked to rate the frequency of certain feelings they have experienced during the past month on a six-point scale from ‘never’ to ‘every day’. The score ranges from 0 to 5, with a higher score indicating higher well-being. The three-factor structure has been confirmed in Dutch, Canadian and American samples [31, 37, 38]. Reliability (Cronbach’s alpha) of the three subscales was .83 for both emotional and psychological well-being and .74 for social well-being in the Dutch study [31]. The instrument had a satisfactory test-retest reliability (.65). The 9-month test-retest reliability for the scales was .46 for emotional well-being, .47 for social well-being and .53 for psychological well-being. The three components had good convergent validity [31].

With the MHC-SF, a person’s subscores can can also determine his or her category of positive mental health [39]. Following Keyes’ diagnostic criteria, someone is described as flourishing when having scored ‘every day’ or ‘almost every day’ at least one time for the three emotional well-being items and at least six times for the 11 social and psychological well-being items. When someone scores ‘never’ or ‘once or twice’ at least once on the emotional well-being items and at least six times on the social and psychological well-being items, he or she is categorized as languishing. Individuals who are neither languishing nor flourishing are described as moderately mentally healthy.

#### Secondary outcomes

All secondary outcomes were previously developed and validated scales. A detailed description of the scales can be found in the study design [27]. The following concepts were measured:

- Resilience: Brief Resilience Scale [40] with six items with a maximum score of five;
- Purpose in life: the Purpose in Life Scale [37, 38, 41], one of the six subscales from Ryff’s Psychological Well-being Scales with five items with scores ranging from 5 to 25;
- Depression: CES-D [32, 33] with 20 items. Scores could reach a maximum of 60, with higher scores indicating more depressive symptoms;
- Health-related quality of life: EQ-5D [29] with five items with a maximum score of 1, indicating full health;
- Loneliness: Loneliness Scale [28] with 11 items, ranging from 0 to 11. The total score can be categorized into four levels: not lonely (0–2), moderately lonely (3–8), severely lonely (9–10), and very severely lonely (11);
- Social participation: items from validated national survey studies, i.e., from the Permanent Onderzoek LeefSituatie (POLS) [42], and the Longitudinal Internet Studies for the Social Sciences (LISS) panel [43]. Measured with three items concerning contact with family, friends and neighbours on a five-point scale from ‘at least once a week’ (1) to ‘rarely or never’ (5), and seven items on activities, such as voluntary work, study, helping others, religious activities or hobbies, with ‘yes’ or ‘no’ as answer options. A score between 0 and 5 was computed, with higher scores indicating higher levels of social participation;
- Health care costs: items from the treatment inventory of costs in patients with psychiatric disorders (TiC-P) [44] with nine items on received health care and six items for other forms of received help during the last 4 weeks. We determined the cost prices for each volume of consumption. To calculate costs, volumes of health care use were multiplied by the cost prices for each volume of care. The standard cost prices were derived from the Dutch costing manual [45–47]. Money spent on the Happiness Route budget was also included in the health care costs.
- Quality adjusted life years (QALYs): QALYs were calculated as the area under the EQ-5D curve, using the Dutch tariffs. It was assumed that the EQ-5D score obtained at the final visit after 9 months was representative for Quality of life at 12 months, hence this value was carried forward in the calculation of QALYs.

#### Treatment satisfaction

Treatment satisfaction was measured with mostly open questions at the end of the second and third questionnaires. After 3 months, participants were asked: (1) How good was your relationship with the counsellor on a scale from 1 to 10 (1 = not good at all, 10 = extremely good)? (2) What is your opinion about the home visits of the counsellor? (3) Did the project have any effects on your life, and if yes, can you briefly describe these effects? After 9 months, participants were asked: (1) What is your opinion about the project? and (2) Did the project have any effects on your life, and if yes, can you briefly describe these effects? Responses were coded negative, neutral/ambivalent (both negative and positive aspects) or positive by the principal investigator.

### Statistical analyses

For all variables, deviations of the normal distribution were tested by converting skewness and kurtosis statistics to Z-scores. Important deviations from normality were defined as − 2.58 < Z-score > 2.58 for either kurtosis or skewness [48]. Baseline characteristics for continuous normally distributed variables were summarized using mean and standard deviation (SD) and compared between the Happiness Route and Customized Care, using t-tests for independent samples. Non-normally distributed variables were summarized using medians and the first and third quartiles and compared between the treatment groups using non-parametric test statistics. Percentages of categorical baseline characteristics were calculated and compared between groups using chi-square statistics. At baseline, there were no significant differences between the two conditions on any of the demographic variables, neither on primary or secondary outcome measures, suggesting a successful randomization.

All analyses of primary and secondary outcomes were performed according to the intention-to-treat principle. All participants were analysed in the condition to which they were randomized. Multiple imputation (MI) by chained equations was performed to replace missing values. In MI, different datasets are generated. The MI procedure was performed in accordance with guidelines for MI with clinical trial data [49], which suggests, as a rule of thumb, that MI should not be applied if the percentage of missing values should exceed the cut-off value of 40%. The missing value percentage in our study did not exceed this percentage of 40%. The imputation model included all primary and secondary outcomes. Forty datasets with imputed plausible values were obtained, with 200 iterations between datasets [50]. The method proposed by Licht [51] was used to obtain pooled *p*-values. In the study protocol, we planned to generate five different datasets. It turned out that the differences were very large between datasets, probably due to the smaller sample size. Therefore, we decided to increase the datasets from 5 to 40. Intention-to-treat analysis was compared to the results obtained in the per-protocol population. The per-protocol population consisted of people who completed all three questionnaires together with the intervention.

Multivariate repeated-measures analysis of variance (MANOVA) was used to compare baseline to follow-up scores at 3 and 9 months on the primary and secondary outcome measures between the Happiness Route and Customized Care, with time and group as fixed factors. At follow-up, standardized mean differences (Cohen’s d) were calculated for all primary and secondary outcomes as the difference between the means of the baseline and follow-up measurements per condition, divided by their pooled standard deviation. To interpret effect sizes, we used the interpretation for psychological and behavioural treatment, where effect sizes of 0 to 0.32 can be interpreted as small, effect sizes from 0.33 to 0.55 are seen as medium, and effects of 0.56–1.2 are considered large [52]. We did not conduct moderator analyses, as originally planned, as the power was too small. All statistical analyses were performed with SPSS 23.

The cost-effectiveness analysis was conducted from a health care perspective and was performed in accordance with the ISPOR Good Research Practices guidelines for cost-effectiveness analysis alongside clinical trials [53]. The incremental net benefit (iNMB) of the Happiness Route relative to the control condition was calculated as i $NMB(WTA)= WTA\ \Delta  \overline{Q}-\Delta  \overline{C}$. Willingness to accept (WTA) is a threshold value that represents the minimum cash amount the decision maker would accept to give up one QALY. $\Delta  \overline{Q}$ and $\Delta  \overline{C}$ refer to the means of the incremental effects in QALYs and incremental costs in euros respectively. An iNMB > 0 indicates that the amount of money saved exceeds the WTA threshold and, in the case of the present study, that the Happiness Route would provide better value for money compared with Customized Care. The probability that iNMB > 0 was estimated for various values of the WTA threshold, using a method based on the central limit theorem proposed by Nixon et al. [54], in which it is assumed that the incremental costs and effects have an approximate bivariate normal distribution. The expected value and variance of iNMB was calculated for various values of the WTA threshold in each of the 40 multiply imputed datasets and the results were pooled using Rubin’s rules. The pooled parameter estimates were then used to construct cost-effectiveness acceptability curves that incorporated uncertainty due to both sampling and imputation error.

## Results

### Reach of participants

Participants had a median age of 60.00 years, ranging from 26 to 89 years; 75 were female, 33 were male. There were six people from other European countries (one each from Germany, Belgium, Spain, Romania, Serbia and the former Yugoslavia), five from Asia (one from Russia, one from Indonesia, and three from Turkey), one from South-America (Suriname) and one from North America (the Antilles). Five people had, besides their Dutch citizenship, another cultural background: French, Indian/German, German, Turkish and Swiss. Three people did not indicate their cultural background. For details on the baseline characteristics of the participants, see Table 1.

**Table 1:** Baseline characteristics of participants

	All (***n*** = 108)	Happiness Route (***n*** = 58)	Control condition (***n*** = 50)
**Age, median in years (Q**_**1**_**, Q**_**3**_**)**^**a**^	60.0 (48.3, 68.0)	59.00 (47.5, 68.0)	61.0 (49.0, 70.3)
**Gender: male**^**b**^**, n (%)**	33 (30.6)	16 (27.6)	17 (34.0)
**Cultural background: Dutch**^**b**^**, n (%)**	92 (85.2)	49 (84.5)	43 (86.0)
**Marital status: married**^**b**^**, n (%)**	17 (15.7)	12 (20.7)	5 (10.0)
**Living situation: alone**^**b**^**, n (%)**	77 (71.4)	36 (62.1)	41 (82.0)
***Daily activities***^***b***^**, n (%)**			
**Paid employment**	5 (4.6)	4 (6.9)	1 (2.0)
**Unemployed/household**	30 (28.2)	15 (25.8)	15 (30.0)
**Disability pension**	41 (38.0)	26 (44.8)	15 (30.0)
**Retired**	23 (21.3)	9 (15.5)	14 (28.0)
**Other (student, daycare, volunteer work, caregiver, hobby)**	9 (8.4)	4 (6.8)	5 (10.0)
***Education***^**b**^**, n (%)**			
**Low**	64 (59.4)	33 (57.1)	31 (62.0)
**Intermediate**	25 (23.6)	13 (23.2)	12 (24.0)
**High**	18 (17.0)	11 (19.6)	7 (14.0)
***Monthly income***^**b**^			
**< €1000 for singles; <€1500 for couples, n (%)**	73 (67.3)	37 (63.2)	36 (72.0)
**< €2000 for singles; <€2500 for couples, n (%)**	35 (32.7)	21 (36.8)	14 (28.0)
**Number of health problems, median (Q**_**1**_**, Q**_**3**_**)**^**a**^	3.00 (2.00, 4.00)	3.00 (2.00, 4.00	3.50 (2.00, 3.502.0)
**Health grade, median (SD)**^**a**^	6.00 (4.6, 7.0)	6.00 (4.0, 7.0)	6.00 (4.9, 7.0)
**Psychotropic drug use, n (%)**^**b**^	49 (45.4)	25 (43.1)	24 (48.0))

^a^No significant differences between intervention and control condition (*t*-test with *p* > .05). ^b^No significant differences between intervention and control condition (χ^2^ -test with *p* > .05)

#### Loneliness

Participants had a median score of 9.01 on the loneliness scale at baseline (Q_1_ = 8.00, Q_3_ = 11.00), which is considered as severely lonely [55]. Participants spent most of their time at home; on average, they spent less than 2 h outside per day, with a median score of 7 days per week inside (Q_1_ = 5.00, Q_3_ = 7.00).

#### Health problems

Participants had a median of 3.00 health problems, with up to 10 diseases, indicating a high level of comorbidity. The most frequent diseases occurring across all health problems were internal medicine diseases (37% of all diseases mentioned, e.g., rheumatic and endocrinological diseases), mental disorders (17.1%, e.g., depression and personality disorder), orthopaedics and accident surgery diseases (18.4%, e.g., arthritis and osteoporosis), neurological diseases (8%, e.g., epilepsy and stroke) and nonspecific symptoms and diseases (9.3%, e.g., concentration problems). Diseases occurring less often (each less than 2.7%) were surgical, urinary system, skin, genetic, otorhinolaryngology, dental and eye diseases. A person could score in several categories and more than once in one category. Only one participant did not report having any health problems, but still had an impaired health-related quality of life.

The mean of health-related quality of life, measured with the Dutch EQ-5D, was 0.45 at baseline. The SD of 0.30 indicated a large variation in health-related quality of life. The mean CES-D score of participants was 26.06 (SD 12.16), where a cut-off score of 16 is indicative of clinically relevant depressive symptomatology [56]. Almost half of the participants (45.4%) took psychotropic drugs; 23.1% took benzodiazepines, 23.1% took antidepressants, 9.3% took sleep-inducing drugs, and 9.3% took antipsychotic drugs.

#### SES

Socio-economic status was low. Only 4.6% had paid work. Almost 60% had a low education and close to 70% had a low income.

#### Well-being

Average well-being was very low, with a total mean score of 1.99 (SD 0.91), on a scale from 0 to 5 [31]. Thirty-one percent of the participants were languishing at the start of the intervention.

### Treatment adherence and completion

**Fig. 1:**
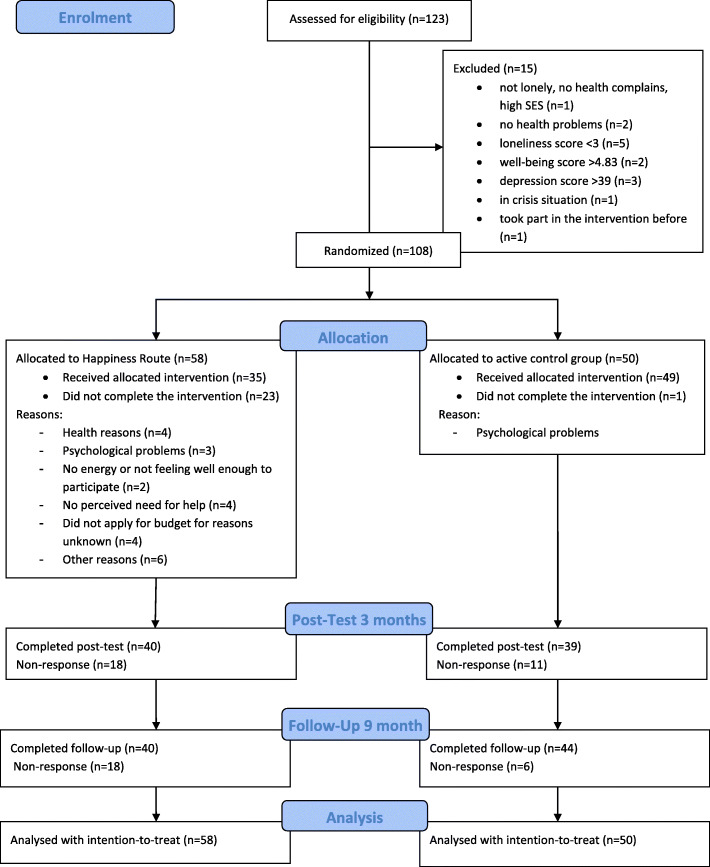
Participant flow diagram

For details about the participant flow, see Fig. 1. Of the 123 candidates assessed for eligibility, 108 participants were randomized. Ten candidates were excluded because they did not have ‘enough’ problems, whereas four had problems that were too severe and one had been a participant of the Happiness Route before. Of the 58 participants randomized to the Happiness Route, 23 did not fully adhere. The non-adherers did not apply for a budget nor did they take part in an activity because, for example, they needed to stop the Happiness Route due to health or psychological problems. There were no significant differences between intervention adherers (*n* = 35) and non-adherers (*n* = 23) of the Happiness Route at baseline, with the exception of ‘daily activity’ (χ^2^_7_ = 14.87, *p* = .04). Intervention adherers were more often unemployed and receivers of a disability pension and less often retired than non-adherers. Of the participants who did adhere to the intervention, 43% followed a course (e.g., a photography course, painting class, or music lessons), 25% made a purchase (e.g., a computer, library card, camera, e-bike, or walking boots), 16% followed a social activity (e.g., bingo, dating, or community centre activities), and 16% started with sports (e.g., gym membership, yoga, or martial arts). The mean of the budget spent was €168.17.

In contrast, only one of the 50 participants included in Customized Care did not fully adhere to the program. Examples of the actions taken in the Customized Care group were more health care, e.g., support by a social psychiatric nurse, ambulant counselling, a social worker at the workplace, (more) domestic help, or extra hours of physiotherapy. One participant wanted less domestic help, which was arranged. Others received more social support, e.g., becoming part of a senior club or going on bus trips with a group, and several participants joined a buddy project. Other forms of help were also organized, e.g., help for the son of a participant, or getting help for financial questions from someone from the participant’s own social network.

The response rate was 100% at baseline, 73.15% at 3-months (79 completers of 108), and 77.78% at 9-months follow-up (84 completers of 108). While at 3 months, the difference in response rate between the participants of the Happiness Route and Customized Care was non-significant (78% vs. 69%; χ^2^_1_ = 1.12, *p* = .29), it was significant at follow-up (88% vs. 69%; χ^2^_1_ = 5.63, *p* = .018). No significant differences at baseline emerged between participants who completed all three measurements (*n* = 76) and non-completers (*n* = 32), with the exception of income (χ^2^_(1)_ = 4.16, *p* = .04) and resilience (t_(106)_ = − 2.08; *p* = .04). More completers (71%) than non-completers (51%) had a low-income level. Completers were less resilient than non-completers (2.51 vs. 2.85). No harm was measured or reported in the two groups during the time period of the study.

### Effectiveness

#### Primary and secondary outcome measures

Table 2 provides the means and standard errors (S.E.) at all three measurement times, as well as tests and effect sizes for the effectiveness of the primary and secondary outcome measures.

**Table 2:** Effects of the Happiness Route, intention-to-treat analysis (all = 108, HR = 58, CC = 50)

		Baseline	3 months	9 months	Condition	Time	Time x condition	Effect size over time (FU)
**Outcome**	Group	*Mean (S.E.)*	*Mean (S.E.)*	*Mean (S.E.)*	*p*	*p*	*p*	*d*
***Primary outcome***								
**MHC-SF total**	HR	2.04 (0.13)	2.07 (0.12)	2.28 (0.12)	.362	.018*	.902	0.38
	CC	1.93 (0.12)	1.96 (0.12)	2.13 (0.13)				0.17
**MHC-SF emo**	HR	2.09 (0.18)	2.40 (0.22)	2.56 (0.20)	.649	.012*	.603	0.32
	CC	2.10 (0.18)	2.19 (0.22)	2.45 (0.21)				0.25
**MHC-SF soc**	HR	1.43 (0.11)	1.57 (0.16)	1.83 (0.16)	.791	.026*	.457	0.25
	CC	1.52 (0.12)	1.54 (0.13)	1.67 (0.13)				0.23
**MHC-SF psy**	HR	2.50 (0.16)	2.33 (0.17)	2.50 (0.16)	.247	.359	.640	0.00
	CC	2.19 (0.15)	2.21 (0.16)	2.34 (0.16)				0.14
***Secondary outcomes***								
**Resilience**	HR	2.59 (0.10)	2.69 (0.17)	2.77 (0.17)	.711	.069	.510	0.17
	CC	2.64 (0.12)	2.83 (0.15)	2.75 (0.14)				0.12
**Purpose in life**	HR	14.95 (0.59)	15.65 (0.83)	15.81 (0.89)	.233	.213	.679	0.15
	CC	14.26 (0.63)	14.66 (0.75)	14.89 (0.78)				0.13
**Depression**	HR	27.05 (1.76)	24.56 (2.29)	21.19 (2.58)	.772	.032*	.169	0.35
	CC	25.04 (1.49)	25.74 (2.08)	23.67 (2.29)				0.10
**Quality of life**	HR	0.45 (0.04)	0.49 (0.07)	0.51 (0.07)	.716	.118	.572	0.13
	CC	0.46 (0.05)	0.54 (0.06)	0.50 (0.06)				0.08
**Loneliness**	HR	8.65 (0.32)	8.51 (0.52)	7.42 (0.74)	.014*	.019*	.486	0.28
	CC	9.41 (0.29)	9.43 (0.39)	8.85 (0.52)				0.19
**Participation**	HR	1.81 (0.21)	1.52 (0.31)	1.47 (0.45)	.456	.251	.376	0.13
	CC	1.90 (0.21)	1,94 (0.26)	1.71 (0.33)				0.10

*FU* Follow-up, *HR* Happiness Route, *CC* Customized Care, *emo* emotional, *soc* social, *psy* psychological; *p < .05

##### Primary outcome

No significant time x group interaction effect was found in the MANOVA analysis of the primary endpoint. However, the total score of the MHC-SF and the subscales of both emotional and social well-being improved significantly over the 9-month period in both groups (*p* < .05). No significant improvement was found in the subscale of psychological well-being. No significant interaction effect between time and condition was found for the total scale, nor the subscales of the MHC-SF. The effect size (Cohen’s d) for the total score of well-being was somewhat higher for the Happiness Route (0.38), compared to Customized Care (0.17). The effect size for the Happiness Route can be considered moderate, whereas the effect size for the control condition is considered small [52]. There were no differences in results when adherers-only to the Happiness Route were compared to adherers in the Customized Care. The general pattern of the results in the per-protocol population was similar to the results of the intention-to-treat analysis (see Additional file 1).

In Table 3, the three mental health categories were used to assess the development of well-being. Across the conditions and across time, there were no significant differences at baseline (χ^2^_2_ = 3.84, *p* = .147). Most participants had moderate mental health, followed by those who were languishing. The percentage of languishers decreased significantly by half, from more than 30% at baseline to less than 16% at follow-up in the Happiness Route (marginal homogeneity test with *p* = .029). The control condition did not change significantly (marginal homogeneity test with *p* = .593).

**Table 3:** Number of participants and percentages (%) in the three mental health categories for completers only

	Mental health category	Happiness Route	Control Condition
**Baseline**	Languishing	19 (32.8%)	14 (28.0%)
	Moderately mentally healthy	32 (55.2%)	34 (68.0%)
	Flourishing	7 (12.1%)	2 (4.0%)
**3-month**	Languishing	14 (36.8%)	12 (27.9%)
	Moderately mentally healthy	20 (52.6%)	39 (69.8%)
	Flourishing	4 (10.5%)	1 (2.3%)
**9-month**	Languishing	6 (15.8%)	10 (23.3%)
	Moderately mentally healthy	27 (71.1%)	32 (74.4%)
	Flourishing	5 (13.2%)	1 (2.3%)

##### Secondary outcomes

Concerning the secondary outcome measures, no significant interaction effect between time and group was found. A significant improvement over time was found for both groups in depression and loneliness (*p* < .05), but not for resilience, purpose in life, health-related quality of life and social participation. The effects tended to be somewhat higher in the Happiness Route than in Customized Care (Cohen’s d for depression was .35 versus .10 and for loneliness .28 versus .19).

### Treatment satisfaction 

#### 3-month

At 3 months, there was a significant difference between the grades the participants of the two conditions gave to their relationship with the counsellor (t_(69)_ = 3.12; *p* = .003). Concerning the open question of how participants experienced the home visits of the counsellor, Happiness Route participants were significantly more likely to be positive (χ^2^_3_ = 14.18, *p* < .01). An example of a positive evaluation of the counsellor in the experimental condition was: ‘Very nice, it helped me a great deal to help me get back on track. It taught me to look after myself, as well. And also to stand up for myself and now decide about a lot of things on my own.’ In the Customized Care condition, 15.4% of the participants evaluated the home visits negatively. An example of a negative evaluation by a participant of the Customized Care condition was: ‘I did not experience that home visit as pleasant. Too much about the past’. When asked to give a judgment on the effects of the intervention, participants of the Happiness Route were significantly more likely to experience positive effects than participants of the control condition (χ^2^_3_ = 11.88, *p* = .008). For more details, see Table 4.

**Table 4:** Treatment satisfaction in the Happiness Route and Customized Care conditions

	Happiness Route	Control Condition	Difference between conditions ***p***-value
**3-month**			
***Grade for counsellor***, mean (SD)	8.49 (1.37)	7.25 (1.95)	.003*
***Evaluation of home visits***, %	*n* = 39	*n* = 39	< .01*
Negative	.0	15.4	
Neutral/ambivalent	2.6	20.5	
Positive	92.3	61.5	
Missing	5.1	2.6	
***Judgment on the effects***, %	*n* = 38	*n* = 39	.008*
No effects	18.4	56.4	
Neutral/ambivalent	15.8	7.7	
Positive	50.0	28.2	
Missing	15.8	7.7	
**9-month**			
***Evaluation of home visits***, %	*n* = 39	*n* = 41	.016*
Negative	10.5	22.0	
Neutral/ambivalent	15.8	26.8	
Positive	71.1	36.6	
Missing	2.6	14.6	
***Judgment on the effects***, %	*n* = 34	*n* = 38	.025*
No effects	26.5	52.6	
Neutral/ambivalent	14.7	13.2	
Positive	56.0	23.7	
Missing	2.9	10.5	

*p < .05

#### 9-month

In the follow-up measurement, participants of the experimental condition were significantly less likely to be negative and more likely to be satisfied with the intervention than the participants of the control condition (χ^2^_3_ = 10.30, *p* = .016). For example, one positive reaction of a Happiness Route participant was: ‘Yes, it certainly has given me the opportunity to go out of the house more, and to come in contact with people more often. I am more active, also by going to a gym. Thank you!’ In the Customized Care group, one of the participants who evaluated the intervention negatively said: ‘Not interesting. I do not see any progress and I find it tiring.’

During follow-up, Happiness Route participants were significantly more likely to describe positive effects than the Customized Care participants (χ^2^_3_ = 9.35, *p* = .025). An example from the Happiness Route was: ‘Enthusiastic, discovered that there are things that I can enjoy again. Being proud of what I do at the course and getting compliments for it.’ A negative evaluation was: ‘No effect, it did not bring any changes. The activity of hiking was something that I already did before’. A percentage of 52.6% of the participants did not experience any effects (e.g., ‘No effect due to minimal guidance.’), and 13.2% were neutral or had mixed feelings about effects*.* A percentage of 23.7% experienced positive effects (e.g., ‘Moved to a very nice house. Even got help from social services. Due to mentioned emotional stimuli, I really started thinking about how I ended up in this bad situation.’

#### Cost-effectiveness

The intervention was associated with both higher costs and fewer QALYs on average. Participants in the Happiness Route gained 0.07 (95% CI, [0.05; 0.11]) less QALYs on average compared with participants in the control group. Additional file 2 describes costs of the intervention and control groups. The average costs for implementing the intervention (i.e., the happiness budget) was €168. Participants in the control group had higher monthly medical care consumption costs throughout the intervention (range of incremental costs = €2.81 - €58.75 per month) and higher (in)formal care and productivity loss related costs (range = €60.83 - €96.91). The average total costs over the 1-year period were €1090 higher for the control group (95% CI, [−€1336–3516]).

**Fig. 2:**
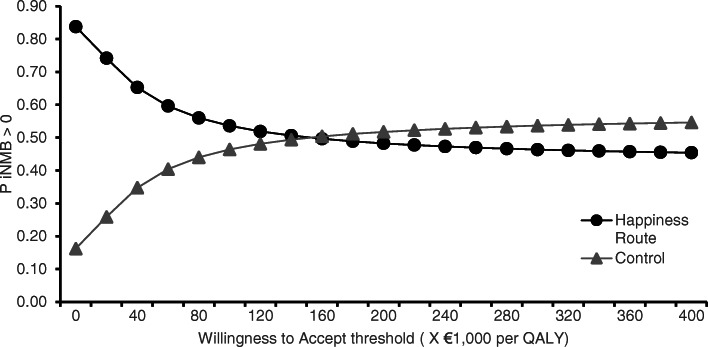
Cost-effectiveness acceptability curves

The results of the cost-effectiveness analysis are summarized in Fig. 2. The mean saved cost per QALY lost (i.e., ICER) was €161,953. If decision makers would only be interested in cost-minimization from a health care perspective (i.e., WTA = €0), our data suggest that there would be an 84% chance that the Happiness Route would be preferable to Customized Care. Uncertainty about the Happiness Route being cost-effective relative to Customized Care increases as a higher monetary value is assigned to the health of participants (i.e., as WTA increases).

## Discussion

No significant differences were found between the Happiness Route and Customized Care on primary and secondary outcomes. Both groups improved significantly in well-being, depression, and loneliness. The standardized effect sizes were somewhat higher for the Happiness Route than for Customized Care and the percentage of languishers decreased significantly from 32.8% at baseline to 15.8% at follow-up for the participants in the Happiness Route, but not in Customized Care. No effects were found for the other secondary outcomes: resilience, purpose in life and health-related quality of life. Participants adhered less often in the experimental than in the control condition. Regarding the level of satisfaction of participants, participants were very satisfied with the Happiness Route. They highly rated the relationship with their counsellor with a grade of 8.5. It is notable that none of the participants were negative about the intervention at 3 months and more than 90% were positive. More than half of them said that they had experienced positive effects both at 3 and 9 months. By addressing positive aspects of life such as values, strengths and passion in the intervention instead of focusing on people’s shortcomings, the happiness-based intervention was received more positively than the problem-based condition. Our cost effectiveness analysis shows that the Happiness Route may save considerable costs, but results in slightly less accrued QALYs compared to Customized Care.

There are at least three possible reasons why an interaction effect was not found. First, power was too low, as The power size calculated beforehand was not reached. Second, the differences between the experimental intervention and the control condition in their approach might have been smaller than expected. For example, some counsellors registered participants in the control condition for a social activity, such as activities in a senior club, or found the participant social support, such as via a buddy project. Furthermore, counsellors looked at the whole picture of care in the Customized Care condition, whereas in everyday practice, different care professionals often approach specific problems from more isolated areas of expertise. Third, the adherence-rate was much lower in the Happiness Route compared to the control condition. This could be explained by the fact that the Happiness Route was more intensive and asked more of the participants than the passive control condition. The Happiness Route required active participation in an activity, often after years of isolation and inactivity on the part of the participant. We have to realize that this could be an immense, perhaps frightening step for many people of this group. When they did persevere, they were very satisfied with the Happiness Route and the effects it had on them. This result corresponds to findings that engaging in a leisure activity that one is intrinsically interested in, such as sports or social activities, is linked to improved well-being [57].

Although there were no differences between the conditions, we found effects for emotional and social well-being, depression, and loneliness over time, which might have been due to regression toward the mean [58], as baseline scores were extremely low. On the other hand, scores for the MHC-SF have proven to be remarkably stable over a period of 9 months in a large panel, representative for the Dutch population [59]. Also, it has been shown to be very difficult to improve loneliness with interventions [60, 61]. Furthermore, only three measures improved over time, whereas resilience, purpose in life and health-related quality also started extremely low, but did not improve significantly over time. These are all strong arguments against the regression toward the mean explanation.

A meta-analysis on PPIs has shown that - even under more controlled conditions and often with non-active control conditions - effect sizes were small, with a standardized mean difference of .34 for subjective well-being, .20 for psychological well-being and .23 for depression [22]. Except for psychological well-being, these findings are in line with the effect sizes that we found.

An explanation for higher satisfaction could be that people generally perceive well-being interventions as less stigmatizing than formal mental health services and, consequently, accept them more easily [61]. Higher satisfaction indicates that a positive psychology approach could be a viable alternative, especially for structurally isolated, vulnerable people who demonstrate care-avoidant behaviour and tend to keep health care that targets their problems at a safe distance [8]. This behaviour is in line with findings from a qualitative study amongst community-dwelling, lonely older people. For the majority of the group, primary care and community-based services addressing their loneliness were seen as neither desirable nor helpful. Yet they considered group-based activities with a shared interest preferable to other forms of support [62]. Being able to offer a more attractive, acceptable and non-stigmatizing approach could help to care for groups that are normally difficult to reach within the formal (mental) health care system [61]. Concerning future research, the intervention could be studied with other target groups at risk for a languishing condition that might be difficult to reach with formal mental health care, for example refugees.

While the cost effectiveness analysis showed that costs were saved in the Happiness Route, willingness to accept thresholds to interpret the results of disinvestment studies have not yet been defined in the Netherlands. However, the willingness to pay for an additional QALY lies between €10,000 and €90,000 and is dependent on the disease burden of the population under consideration. Using these threshold values, the estimated savings of €161,953 per QALY would suggest that the Happiness Route is an acceptable intervention. It should be noted, however, that a disparity exists between the willingness to pay and the willingness to accept; people are generally willing to pay a lower amount of money to acquire one QALY compared with the amount they would minimally want to receive before they are willing to forego 1 QALY [62].

Although recruitment proved to be difficult, a very vulnerable group was reached, which makes our target group especially interesting. The group we included was even more vulnerable than expected based on the inclusion criteria. On average, participants were severely lonely, with a high comorbidity of diseases, serious depressive symptoms and low levels of well-being as compared to the Dutch population. In fact, participants scored almost twice as high as the group in the Dutch population with the highest average loneliness scores [28]. Comparable to patients with moderate depressive symptoms [57], the participants’ health-related quality of life was seriously impaired, with a score that was almost half as low as a representative sample of the Dutch population [63]. Well-being had a low total mean score of 1.99 (SD 0.91) on the MHC-SF, which is more than a standard deviation below the mean of 2.98 (SD 0.85) for the normal Dutch population [31]. While only 5% of the Dutch population languish, 31% of the participants were languishing at the start of the intervention [64]. The yearly mean health costs they produced at baseline were around €8000. Indeed, their high level of suffering and care consumption show that they are an important group to pay attention to and study.

### Limitations and recommendations

Our study had a couple of limitations that have to be kept in mind when interpreting the results. First of all, the study was underpowered. The study had under-recruited and could not reach the sample size that was calculated in the power analysis. Additional to the problems with inclusion, there was a high number of people who did not complete the intervention and who did not complete all questionnaires, both leading to a serious problem with power. Therefore, it is possible that not finding a difference between the experimental and control condition was due to insufficient power, rather than equivalence of the interventions. Accordingly, a possible reason that we did not find an interaction effect between time and group could be that the study was underpowered.

Furthermore, recruitment turned out to be very difficult. First, the ‘invisibility’ of the target group of lonely people could have hindered finding candidates. Being socially isolated and thus having no contact with the outside world makes these people very difficult to detect. As Machielse states, structurally socially isolated people are invisible to society [16]. Second, the fact that the intervention was part of a study with a control condition could have played a role in the limited amount of applications. Intermediaries sometimes wanted to ‘protect’ their clients from the possible stress a research project might expose them to (e.g. filling in long questionnaires), as well as from the chance that they could be randomized to the control condition. Future studies working with intermediaries should be aware of the concerns they might have regarding research and be prepared to adequately and proactively respond to those concerns.

Only 12% of the participants that have been applied for participation had to be excluded, indicating that the intermediaries knew quite well who was qualified for the study. However, the extreme scores of the participants could be a sign that the intermediaries had a picture of the target group that was too extreme. For example, candidates were allowed to take part with a loneliness score of 3, while the mean score at baseline of the included participants was around 9 (with 11 as the highest possible score). These high loneliness scores indicate that the intermediaries seemed to have sought out people who were extremely lonely, and as such, had a distortedpicture of how vulnerable the target group had to be – a possible disadvantage when using sampling by referral. This misperception on the part of the intermediaries could have impeded recruitment, as 28% of the Dutch population is moderately lonely and only 4% is severely or extremely lonely [28]. For future studies, we recommend emphasizing that moderate levels of loneliness also indicate that someone could be applied for the study. Although the real-life setting held limitations for recruitment, it also meant that the external validity of this study was good, as it took place in the field and the recruitment as well as the intervention were conducted as usually practiced.

It is interesting that, while there were no significant differences in effect between groups when measured quantitatively, participants from the Happiness Route, compared to their counterparts in Customized Care, were significantly more likely to describe experiencing positive effects when responding to the open questions, both directly after the intervention and 6 months after home visits had stopped. This disparity could mean that the questionnaire was less sensitive to change than the participants’ own perceptions. Nevertheless, this possible limitation demonstrates the added value of using open questions to validated questionnaires.

Moreover, we did not compare the amount of contact that was given between the two conditions. It is likely that participants of the Happiness Route received more visits than people in the control condition, and this was not taken into account in the analysis. The decision to not level the two conditions concerning the number of sessions was due to the fact that one important aspect of the Happiness Route is the program’s personalized fit to the individual’s needs. While one participant might need only two sessions, another might need twice as much time to discover their passion. We gave the counsellors the freedom to fit the number of sessions (within certain limits) to each individual participant.

Due to the small sample size, we could not conduct any moderator or mediator analysis, which would have been relevant to differentiate for whom the intervention works best. Therefore, we could also not take into account that in the control condition, all counsellors were professionals, while in the Happiness Route, counsellors were both professionals and volunteers. The reason for this difference was that we wanted to stay as close as possible to the traditional health care in the control condition, which meant using only professionals in that condition. We do not expect that there were large differences between the counselling provided by professionals and volunteers. The Happiness Route was a new intervention for both professionals and volunteers and they were trained in the exact same manner, thus both started at the same level. Furthermore, the volunteers often had many years of experience in the health care sector or even worked in the sector, but volunteered for this project.

A final challenge of this study was the difficulty in clearly differentiating the working ‘ingredients’ of the intervention. A future study could use different conditions, e.g., comparing the original intervention with a condition that only offers the rapportbuilding part, a condition where participants receive the money without counsellor support, and a condition where participants receive support, but no money. Furthermore, an analysis of treatment integrity, for example, based on audio-visual recordings, would allow for more in-depth insights into which parts of the intervention were actually used as intended.

## Conclusions

This was the first study that evaluated a PPI for people with an accumulation of risk factors for low well-being in a practice-based multi-site trial. The findings suggest that the Happiness Route is as effective as the current, problem-based care in the Netherlands for a very vulnerable part of the population. The Happiness Route might be better to help languishing people to become moderately mentally healthy, which can lead to great individual and social benefits [65]. Participants who completed the intervention evaluated it more positively than problem-based care. Our results should be viewed in light of the fact that we were not able to include the planned number of participants. Nevertheless, as the participants benefitted from the Happiness Route and the intervention proved to be cost-effective, it seems to be an acceptable alternative intervention for vulnerable people that fits current developments in health and social care.

## Supplementary information

**Additional file 1.** Per-protocol analysis.

**Additional file 2.** Monthly medical costs by treatment group.

## Data Availability

The datasets generated and analysed during the current study are not publicly available because it could compromise the individual privacy of the participants, but are available from the corresponding author on reasonable request.

## Abbreviations

CES-D: Center for Epidemiology Depression Scale
EQ-5D: EuroQol 5D
iNMB: Incremental net benefit
MHC-SF: Mental Health Continuum-Short Form
MI: Multiple imputation
POLS: Permanent Onderzoek LeefSituatie
PPIs: Positive psychology interventions
SD: Standard deviation
TiC-P: Treatment inventory of Costs in Patients with psychiatric disorders
QALYs: Quality adjusted life years

## References

[1] Mechanic D, Tanner J. Vulnerable people, groups, and populations: societal view. Health Aff. 2007. 10.1377/hlthaff.26.5.1220. 10.1377/hlthaff.26.5.1220 17848429

[2] Keyes CLM (2007). Promoting and protecting mental health as flourishing. A complementary strategy for improving national mental health. Am Psychol.

[3] Keyes CLM, Shapiro AD, Brim OG, Ryff CD, Kessler RC (2004). Social well-being in the United States: a descriptive epidemiology. How healthy are we? A national study of well-being at midlife.

[4] Diener E, Suh EM, Lucas RE, Smith HL (1999). Subjective well-being: three decades of progress. Psychol Bull.

[5] Mangelli L, Gribbin N, Büchi S, Allard S, Sensky T (2002). Psychological well-being in rheumatoid arthritis: relationship to ‘disease’ variables and affective disturbance. Psychother Psychosom.

[6] Ryff CD, Singer BH (2008). Know thyself and become what you are: a eudaimonic approach to psychological well-being. J Happiness Stud.

[7] Golden J, Conroy RM, Bruce I, Denihan A, Greene E, Kirby M, Lawlor BA (2009). Loneliness, social support networks, mood and wellbeing in community-dwelling elderly. Int J Geriatr Psychiatry.

[8] Pinquart M, Sörensen S (2000). Influences of socioeconomic status, social network, and competence on subjective well-being in later life: a meta-analysis. Psychol Aging.

[9] Cole K (2006). Wellbeing, psychological capital, and unemployment: an integrated theory.

[10] Masi CM, Chen H-Y, Hawkley LC, Cacioppo JT (2011). A meta-analysis of interventions to reduce loneliness. Personal Soc Psychol Rev.

[11] Holt-Lunstad J, Smith TB, Baker M, Harris T, Stephenson D (2015). Loneliness and social isolation as risk factors for mortality: a meta-analytic review. Perspect Psychol Sci.

[12] Flaskerud JH, Winslow BJ (1998). Conceptualizing vulnerable populations health-related research. Nurs Res.

[13] Valtorta NK, Kanaan M, Gilbody S, Ronzi S, Hanratty B (2016). Loneliness and social isolation as risk factors for coronary heart disease and stroke: systematic review and meta-analysis of longitudinal observational studies. Heart..

[14] Lorant V, Croux C, Weich S, Deliège D, Mackenbach J, Ansseau M (2007). Depression and socio-economic risk factors: 7-year longitudinal population study. Br J Psychiatry.

[15] Van der Plaats JJ (2002). Eindrapportage Zorg in Beeld Verlicht.

[16] Machielse A (2011). Sociaal isolement bij ouderen: een typologie als richtlijn voor effectieve interventies. JSI..

[17] Seligman MEP, Csikszentmihalyi M (2000). Positive psychology. An introduction. Am Psychol.

[18] Barry MM, Jenkins R (2007). Implementing mental health promotion.

[19] Herrman H, Saxena S, Moodie R. Promoting mental health: concepts, emerging evidence, practice: a report of the World Health Organization, Department of Mental Health and Substance Abuse in collaboration with the Victorian Health Promotion Foundation and the University of Melbourne. World Health Organization. Geneva: World Health Organization; 2005.

[20] Lyubomirsky S, King L, Diener E (2005). The benefits of frequent positive affect: does happiness lead to success?. Psychol Bull.

[21] Weiss LA, Westerhof GJ, Bohlmeijer ET. Can we increase psychological well-being? The effects of interventions on psychological well-being: a meta-analysis of randomized controlled trials. PLoS One. 2016. 10.1371/journal.pone.0158092. 10.1371/journal.pone.0158092 PMC4915721 27328124

[22] Bolier J, Haverman M, Westerhof GJ, Riper H, Smit F, Bohlmeijer E. Positive psychology interventions: a meta-analysis of randomized controlled studies. BMC Public Health. 2013. 10.1186/1471-2458-13-119. 10.1186/1471-2458-13-119 PMC3599475 23390882

[23] Sin NL, Lyubomirsky S (2009). Enhancing well-being and alleviating depressive symptoms with positive psychology interventions: a practice-friendly meta-analysis. J Clin Psychol.

[24] Grumbach K (2003). Chronic illness, comorbidities, and the need for medical generalism. Ann Fam Med.

[25] Francissen AA, Wezenberg EJ, Westerhof GJ (2010). De gevolgen van geluk. Achtergronden en toekomst van het geluksbudget.

[26] Weiss LA, Kedzia S, Francissen A, Søraker J, Van der Rijt JW, de Boer J, Wong PH, Brey P (2015). Westerhof GJ. Improving the health care sector with a happiness-based approach. The case of the Happiness Route. Well-Being in contemporary society.

[27] Weiss LA, Westerhof GJ, Bohlmeijer ET. Nudging socially isolated people towards well-being with the 'Happiness Route': design of a randomized controlled trial for the evaluation of a happiness-based intervention. Health Qual Life Outcomes. 2013. 10.1186/1477-7525-11-159. 10.1186/1477-7525-11-159 PMC3848856 24053566

[28] De Jong GJ, van Tilburg T (1999). Manual of the loneliness scale.

[29] Brooks R (1996). EuroQol: the current state of play. Health Policy.

[30] Centraal Bureau voor de Statistiek, Sociaal en Cultureel Planbureau (2013). Armoedesignalement 2013.

[31] Lamers SMA, Westerhof GJ, Bohlmeijer ET, ten Klooster PM, Keyes CLM (2011). Evaluating the psychometric properties of the mental health continuum-short form (MHC-SF). J Clin Psychol.

[32] Radloff LS (1977). The CES-D scale: a self-report depression scale for research in the general population. Appl Psychol Meas.

[33] Bouma J, Ranchor AV, Sanderman R, van Sonderen E (1995). Het meten van symptomen van depressie met de CES-D. Een handleiding. 2nd ed.

[34] Parks AC, Biswas-Diener R, Kashdan TB, Ciarrochi J (2013). Positive interventions: past, present, and future. Mindfulness, acceptance, and positive psychology. The seven foundations of well-being.

[35] Ryan RM, Deci EL (2000). Self-determination theory and the facilitation of intrinsic motivation, social development, and well-being. Am Psychol.

[36] Hermans D, van de Putte J (2004). Cognitieve gedragstherapie bij depressie.

[37] Ryff CD (1989). Happiness is everything, or is it? Explorations on the meaning of psychological well-being. J Pers Soc Psychol.

[38] Ryff CD, Keyes CLM (1995). The structure of psychological well-being revisited. J Pers Soc Psychol.

[39] Keyes CL (2009). Brief description of the mental health continuum short form (MHC-SF).

[40] Smith BW, Dalen J, Wiggins K, Tooley E, Christopher P, Bernard J (2008). The brief resilience scale: assessing the ability to bounce back. Int J Behav Med.

[41] Steverink N, Westerhof GJ, Bode C, Dittmann-Kohli F (2001). Dutch aging survey: onderzoekdesign en instrumenten. Een onderzoek naar de leefsituatie en het welbevinden van mensen vanaf 40 jaar.

[42] Centraal Bureau voor de Statistiek (2005). Permanent onderzoek leefsituatie.

[43] Scherpenzeel A (2009). Start of the LISS panel. Sample and recruitment of a probability-based internet panel.

[44] Hakkaart-van Roijen L, Van Straten A, Donker M, Tiemens B (2002). Handleiding Trimbos/iMTA questionnaire for costs associated with psychiatric illness (TiC-P).

[45] Hakkaart-van RL (2002). Handleiding TiC-P. Vragenlijst voor zorggebruik en productieverliezen bij psychische aandoeningen.

[46] Hakkaart-van Roijen L, Hoeijenbos M, Regeer E, Ten Have M, Nolen W, Veraart C, Rutten F (2004). The societal costs and quality of life of patients suffering from bipolar disorder in the Netherlands. Acta Psychiatr Scand.

[47] Hoefman RJ, Van Exel NJA, Brouwer WBF (2013). iVICQ. iMTA valuation of informal care questionnaire.

[48] Ghasemi A, Zahediasl S (2012). Normality tests for statistical analysis: a guide for non-statisticians. Int J Endocrinol Metab.

[49] Jakobsen JC, Gluud C, Wetterslev J, Winkel P. When and how should multiple imputation be used for handling missing data in randomised clinical trials - a practical guide with flowcharts. BMC Med Res Methodol. 2017. 10.1186/s12874-017-0442-1. 10.1186/s12874-017-0442-1 PMC5717805 29207961

[50] White IR, Royston P, Wood AM (2011). Multiple imputation using chained equations: issues and guidance for practice. Stat Med.

[51] Licht C (2010). New methods for generating significance levels from multiply-imputed data.

[52] Lipsey MW, Wilson DB (1993). The efficacy of psychological, educational, and behavioral treatment. Confirmation from meta-analysis. Am Psychol.

[53] Ramsey SD, Willke RJ, Glick H, Reed SD, Augustovski F, Jonsson B, Briggs A, Sullivan SD (2015). Cost-effectiveness analysis alongside clinical trials II—an ISPOR good research practices task force report. Value Health.

[54] Nixon RM, Wonderling D, Grieve RD (2010). Non-parametric methods for cost-effectiveness analysis: the central limit theorem and the bootstrap compared. Health Econ.

[55] Van Tilburg TG, De Jong Gierveld J (1999). Cesuurbepaling van de eenzaamheidsschaal. 1999. Tijdschr Gerontol Geriatr.

[56] Lewinsohn PM, Seeley JR, Roberts RE, Allen NB (1997). Center for epidemiologic studies depression scale (CES-D) as a screening instrument for depression among community-residing older adults. Psychol Aging.

[57] Sobocki P, Ekman M, Ågren H, Krakau I, Runeson B, Mårtensson B, Jönsson B (2007). Health-related quality of life measured with EQ-5D in patients treated for depression in primary care. Value Health.

[58] Barnett AG, van der Pols JC, Dobson AJ (2005). Regression to the mean: what it is and how to deal with it. Int J Epidemiol.

[59] Lamers SMA, Glas CAW, Westerhof GJ, Bohlmeijer ET (2012). Longitudinal evaluation of the mental health continuum-short form (MHC-SF). Measurement invariance across demographics, physical illness and mental illness. Eur J Psychol Assess.

[60] Fokkema T, van Tilburg T (2006). Aanpak van eenzaamheid: helpt het?. Een vergelijkend effect- en procesevaluatie-onderzoek naar interventies ter voorkoming en vermindering van eenzaamheid onder ouderen Den Haag: NIDI.

[61] Findlay RA (2003). Interventions to reduce social isolation amongst older people: where is the evidence?. Ageing Soc.

[62] O'Brien BJ, Gertsen K, Willan AR, Faulkner LA (2002). Is there a kink in consumers' threshold value for cost-effectiveness in health care?. Health Econ.

[63] Stolk E, Krabbe P, Busschbach J, Busschbach J, Rabin R, De Charro F (2007). Using the internet to collect EQ-5D norm scores: a valid alternative. 24th scientific plenary meeting of the EuroQol group - proceedings.

[64] Westerhof GJ, Keyes CLM (2008). Geestelijke gezondheid is meer dan de afwezigheid van geestelijke ziekte. MGV..

[65] Keyes CL (2010). The next steps in the promotion and protection of positive mental health. Can J Nurs Res.

