# Supplementary material for: The long and winding road to happiness: A randomized controlled trial and cost-effectiveness analysis of a positive psychology intervention for lonely people with health problems and a low socio-economic status
Source: Health Qual Life Outcomes. 2020 Jun 2;18:162. doi: 10.1186/s12955-020-01416-x (PMC7268769; doi:10.1186/s12955-020-01416-x)
Supplement: Supplementary file 1 — Additional file 1. Per-protocol analysis. [file 12955_2020_1416_MOESM1_ESM.docx]

Additional file 1

Per-protocol analysis (all=64, HR=27, CC=37)

|  | | **Baseline** | **3 months** | **9 months** | **Condition** | **Time** | **Time x condition** |
| --- | --- | --- | --- | --- | --- | --- | --- |
| **Outcome** | Group | *Mean (S.E.)* | *Mean (S.E.)* | *Mean (S.E.)* | *p* | *p* | *p* |
| ***Primary outcome*** | | | | | | | |
| **MHC-SF total** | HR | 1.93 (0.15) | 2.12 (0.15) | 2.35 (0.16) | .52 | .008* | .58 |
|  | CC | 1.95 (0.15) | 1.98 (0.15) | 2.16 (0.16) |  |  |  |
| **MHC-SF emo** | HR | 1.90 (0.20) | 2.40 (0.22) | 2.59 (0.21) | .95 | .003* | .226 |
|  | CC | 2.13 (0.19) | 2.25 (0.21) | 2.39 (0.21) |  |  |  |
| **MHC-SF soc** | HR | 1.43 (0.15) | 1.61 (0.14) | 1.81 (0.17) | .10 | .13 | .664 |
|  | CC | 1.54 (0.15) | 1.54 (0.14) | 1.68 (0.16) |  |  |  |
| **MHC-SF psy** | HR | 2.38 (0.19) | 2.32 (0.19) | 2.59 (0.19) | .37 | .16 | .80 |
|  | CC | 2.19 (0.15) | 2.21 (0.16) | 2.34 (0.16) |  |  |  |
| ***Secondary outcomes*** | | | | | | | |
| **Resilience** | HR | 2.57 (0.13) | 2.69 (0.12) | 2.75 (0.12) | .47 | .11 | .99 |
|  | CC | 2.48 (0.12) | 2.62 (0.11) | 2.65 (0.12) |  |  |  |
| **Purpose in life** | HR | 15.12 (0.73) | 15.77 (0.67) | 16.27 (0.69) | .20 | .10 | .90 |
|  | CC | 14.69 (0.72) | 14.97 (0.66) | 15.43 (0.68) |  |  |  |
| **Depression** | HR | 28.87 (1.90) | 25.31 (1.99) | 22.75 (2.02) | .96 | .06 | .098 |
|  | CC | 25.39 (1.90) | 26.72 (1.99) | 24.72 (2.02) |  |  |  |
| **Quality of life** | HR | 0.40 (0.06) | 0.46 (0.05) | 0.47 (0.06) | .82 | .28 | .26 |
|  | CC | 0.47 (0.05) | 0.52 (0.05) | 0.43 (0.06) |  |  |  |
| **Loneliness** | HR | 8.72 (0.40) | 8.55 (0.38) | 7.79 (0.44) | .03* | .03* | .61 |
|  | CC | 9.27 (0.39) | 9.58 (0.37) | 8.84 (0.43) |  |  |  |
| **Participation** | HR | 1.91 (0.26) | 1.94 (0.25) | 1.71 (0.27) | .54 | .29 | .89 |
|  | CC | 2.17 (.25) | 2.09 (0.25) | 1.83 (0.26) |  |  |  |

Notes. FU = Follow-up; HR = Happiness Route; CC = Customized Care; emo = emotional; soc =social; psy = psychological; *p<.05;
