# Supplementary material for: The long and winding road to happiness: A randomized controlled trial and cost-effectiveness analysis of a positive psychology intervention for lonely people with health problems and a low socio-economic status
Source: Health Qual Life Outcomes. 2020 Jun 2;18:162. doi: 10.1186/s12955-020-01416-x (PMC7268769; doi:10.1186/s12955-020-01416-x)
Supplement: Supplementary file 2 — Additional file 2. Monthly medical costs by treatment group. [file 12955_2020_1416_MOESM2_ESM.docx]

Additional File 2

Monthly medical costs by treatment group

|  | **Happiness Route** | | | **Control group** | | | **Incremental costs (€)** |
| --- | --- | --- | --- | --- | --- | --- | --- |
|  | **n** | **Mean (€)** | **SD (€)** | **n** | **Mean (€)** | **SD (€)** |  |
| **Medical care consumption Baseline** | 57 | 244.53 | \| 34.06 \| \| --- \| | 50 | \| 256.66 \| \| --- \| | 33.15 | -12.13 |
| **Medical care consumption Month 3** | 57 | 217.64 | \| 42.66 \| \| --- \| | 50 | \| 220.45 \| \| --- \| | 39.93 | -2.81 |
| **Medical care consumption Month 9** | 57 | 231.30 | \| 42.86 \| \| --- \| | 50 | \| 290.06 \| \| --- \| | 59.26 | -58.75 |
| **(In)formal care Baseline** | 57 | 376.87 | \| 90.76 \| \| --- \| | 50 | \| 437.70 \| \| --- \| | 99.70 | -60.83 |
| **(In)formal care month 3** | 57 | 286.17 | \| 62.95 \| \| --- \| | 50 | \| 378.79 \| \| --- \| | 89.60 | -92.62 |
| **(In)formal care month 9** | 57 | 211.64 | 54.10 | 50 | \| 308.54 \| \| --- \| | 61.08 | -96.91 |
| **Intervention costs** | 57 | 168.18 | 28.07 | 50 | \| 0.00 \| \| --- \| | 0.00 | 168.18 |
